# P-661. Impact of Optimal β-lactam Treatment on the Outcomes of Patients with *Pseudomonas Aeruginosa* Pneumonia

**DOI:** 10.1093/ofid/ofae631.858

**Published:** 2025-01-29

**Authors:** Ghayur Saeed, Lloyd G Clarke, Sunish Shah, Erin K McCreary, Ryan K Shields

**Affiliations:** University of Pittsburgh, Pittsburgh, Pennsylvania; University of Pittsburgh, Pittsburgh, Pennsylvania; Antibiotic Management Program, UPMC Presbyterian Hospital, Pittsburgh, PA, Pittsburgh, Pennsylvania; University of Pittsburgh Medical Center, Pittsburgh, PA; University of Pittsburgh, Pittsburgh, Pennsylvania

## Abstract

**Background:**

*P. aeruginosa* is the most common cause of nosocomial pneumonia and accounts for a large proportion of antibiotic use in the ICU. Despite this, there are limited comparative-effectiveness data for β-lactam agents. Our objective was to compare outcomes of Pseudomonal pneumonia treated with piperacillin-tazobactam (TZP), cefepime (FEP) or meropenem (MEM).

**Figure:**
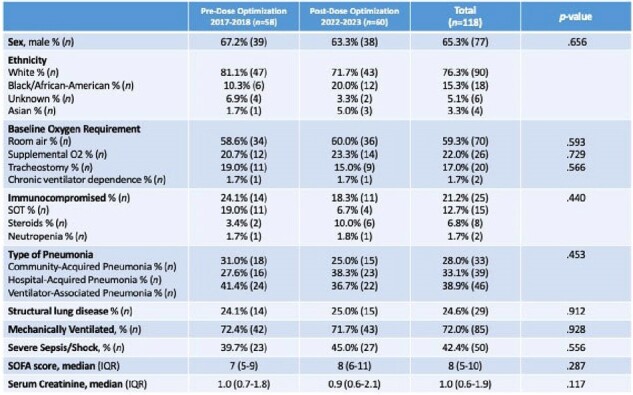

**Methods:**

We performed a retrospective cohort study of patients who met clinical criteria for pneumonia before (2017-2018) and after (2022-2023) dose optimization protocols were established.The primary outcome was 28-day all-cause mortality with secondary outcomes including 28-day clinical response, 90-day recurrence and development of new β-lactam resistance.

**Figure:**
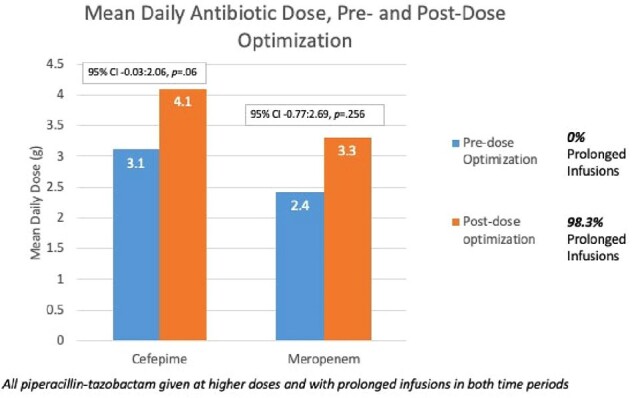

**Results:**

In preliminary analyses, 118 patients were included; baseline characteristics shown in Figure 1. At diagnosis, 72% required mechanical ventilation and 42% had severe sepsis/shock. TZP was giving as a prolonged infusion (≥3h) throughout the study; 98.3% of patients received FEP or MEM as prolonged infusions after dose optimization, compared to 0% before (Figure 2).

Clinical outcomes stratified by definitive treatment are shown in Figure 3. Mortality rates were numerically higher in patients treated with TZP (28.6%) compared to FEP or MEM (22.6%). Rates of treatment emergent resistance ranged from 28.9 – 47.1% across agents. Clinical response rates did not vary across patients before or after dose optimization (Figure 4); however, the 28-day mortality rates was 4.3% lower post-dose optimization of FEP and MEM. Across all patients, 24% died within 28 days of treatment initiation. Among those who survived (n=90), 26% developed recurrent pneumonia or bacteremia within 90 days.

**Figure:**
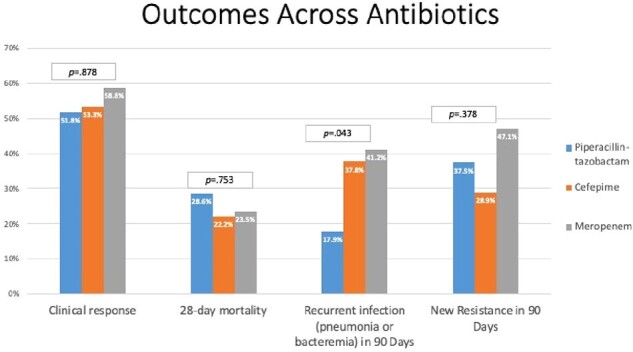

**Conclusion:**

Our preliminary data suggests that outcomes did not vary significantly between TZP, FEP, or MEM for treatment of *P. aeruginosa* pneumonia; however, some notable trends were identified, including higher mortality among patients who received TZP and lower mortality among patients receiving optimized doses of FEP or MEM. These data support further investigation through multicenter studies that include a larger number of patients. Consistent with prior reports, recurrent infections and treatment-emergent resistance are common challenges in managing *P. aeruginosa* pneumonia.

**Figure:**
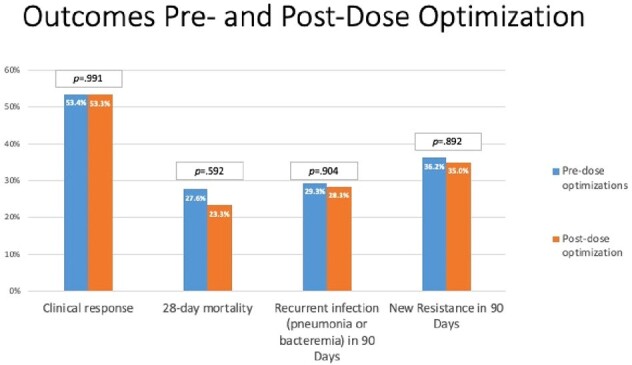

**Disclosures:**

**Erin K. McCreary, PharmD**, Abbvie: Advisor/Consultant|Basilea: Advisor/Consultant|Ciadara: Advisor/Consultant|Entasis: Advisor/Consultant|Ferring: Advisor/Consultant|GSK: Advisor/Consultant|GSK: Honoraria|Melinta: Advisor/Consultant|Merck: Advisor/Consultant|Pfizer: Honoraria|Shionogi: Advisor/Consultant|Shionogi: Honoraria **Ryan K. Shields, PharmD, MS**, Allergan: Advisor/Consultant|Cidara: Advisor/Consultant|Entasis: Advisor/Consultant|GSK: Advisor/Consultant|Melinta: Advisor/Consultant|Melinta: Grant/Research Support|Menarini: Advisor/Consultant|Merck: Advisor/Consultant|Merck: Grant/Research Support|Pfizer: Advisor/Consultant|Roche: Grant/Research Support|Shionogi: Advisor/Consultant|Shionogi: Grant/Research Support|Utility: Advisor/Consultant|Venatorx: Advisor/Consultant|Venatorx: Grant/Research Support

